# Evolutionary Beamforming Optimization for Radio Frequency Charging in Wireless Rechargeable Sensor Networks

**DOI:** 10.3390/s17081918

**Published:** 2017-08-20

**Authors:** Ke-Han Yao, Jehn-Ruey Jiang, Chung-Hsien Tsai, Zong-Syun Wu

**Affiliations:** Department of Computer Science and Information Engineering, National Central University, Taoyuan City 32001, Taiwan; falcoyao@gmail.com (K.-H.Y.); chtsai@ndu.edu.tw (C.-H.T.); 105582002@cc.ncu.edu.tw (Z.-S.W.)

**Keywords:** RF charging, beamforming, antenna array, evolutionary algorithm, evolution strategy

## Abstract

This paper investigates how to efficiently charge sensor nodes in a wireless rechargeable sensor network (WRSN) with radio frequency (RF) chargers to make the network sustainable. An RF charger is assumed to be equipped with a uniform circular array (UCA) of 12 antennas with the radius *λ*, where *λ* is the RF wavelength. The UCA can steer most RF energy in a target direction to charge a specific WRSN node by the beamforming technology. Two evolutionary algorithms (EAs) using the evolution strategy (ES), namely the Evolutionary Beamforming Optimization (EBO) algorithm and the Evolutionary Beamforming Optimization Reseeding (EBO-R) algorithm, are proposed to nearly optimize the power ratio of the UCA beamforming peak side lobe (PSL) and the main lobe (ML) aimed at the given target direction. The proposed algorithms are simulated for performance evaluation and are compared with a related algorithm, called Particle Swarm Optimization Gravitational Search Algorithm-Explore (PSOGSA-Explore), to show their superiority.

## 1. Introduction

Radio frequency (RF) charging adopts RF waves as the medium to deliver energy through the air [1,2,3]. It is one type of wireless charging technology with the advantage to eliminate the troubles of connecting cables to devices and replacing batteries. RF chargers can be applied to simultaneously charge many devices equipped with RF energy harvesters to make device sustainable. Typical devices to be charged include wearable and implanted healthcare gadgets in wireless body area networks (WBANs) [4], and sensor nodes in wireless rechargeable sensor networks (WRSNs) [5,6,7,8,9,10].

A WRSN consists of many sensor nodes and a few sink nodes to provide services such as surveillance, environment monitoring, and home security. A WRSN sensor node has sensing modules to sense physical phenomena (e.g., temperature, humidity, and light intensity) and a wireless communication module to deliver/forward the sensed data to a sink node via multi-hop wireless transmission. It also has a harvester that harvests energy from energy sources in order to replenish its battery, which makes it sustainable. Typical energy sources include solar power, thermal power, wind power, and RF power. Several studies [5,6,7,8,9,10] assume that a WRSN sensor node is equipped with an RF energy harvester to harvest the energy emitted by RF chargers. The studies in [5,6,7] focus on the deployment optimization of RF chargers equipped with directional antennas. The studies in [8,9,10] investigate how to minimize the total time to charge all sensor nodes with mobile chargers equipped with directional or omni-directional antennas. 

An RF charger generates radio waves by applying alternating currents (AC) to an antenna to produce time-varying electromagnetic fields that radiate outward from the antenna to distribute energy. Directional antennas can be used to concentrate energy within a specific range for increasing charging distance and charging efficiency. For example, the Powercast TX91501-3W-ID wireless charger [11] uses a directional patch antenna to have an effective charging distance of 3–5 m and an effective charging angle of 30°–45° between itself and the Powercast P2110-EVAL-02 harvester [12]. The directional antenna can be moved to point to a harvester and deliver most of its energy to it. However, the movement is time-consuming and is even impossible in some cases. On the contrary, the antenna array can be used to swiftly adjust the effective charging region by the beamforming technology without moving the antenna. The existing research results [5,6,7,8,9,10] assume RF chargers are attached with either directional antennas or omnidirectional antennas. To the best of our knowledge, no research has yet investigated applying antenna arrays to RF chargers for charging WRSN sensor nodes. This motivates us to investigate attaching an antenna array to the RF charger to make it efficiently charge WRSN sensor nodes.

**Figure 1:**
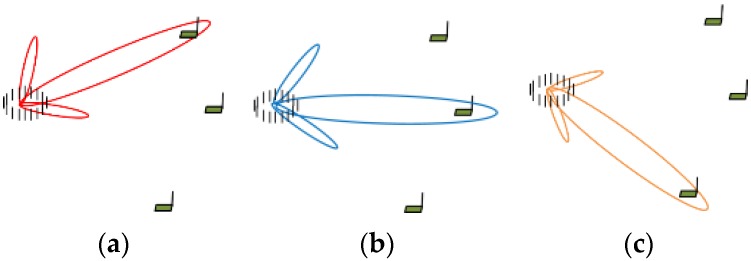
An antenna array can steer the strongest energy beams to aim at different directions, e.g., directions to the (**a**) upper right sensor; (**b**) rightmost sensor; and (**c**) lower right sensor.

**Figure 2:**
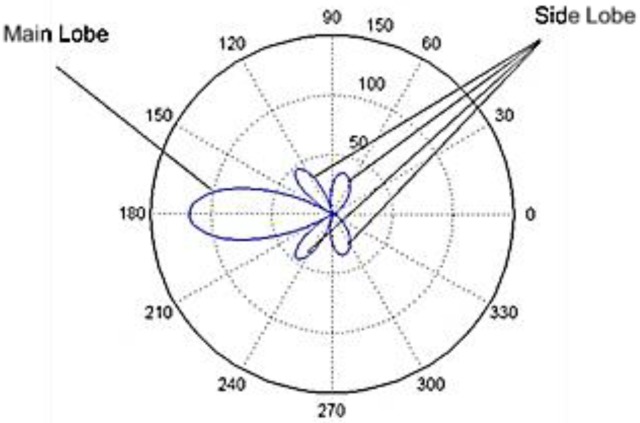
The beamforming radiation pattern or beam pattern showing the main lobe and side lobes.

An antenna array is a set of multiple antennas, called elements, which are connected together and arranged into different geometrical configurations, such as linear, planar and circular structures [13]. The RF waves radiated by each element are constructively and destructively combined together so that the power radiated in the target direction is enhanced, and the power radiated in other directions is reduced. This is called beamforming, and can be achieved by varying the phase and the amplitude of signals fed to each element of the array. For example, Figure 1 shows a circular antenna array that generates the strongest beam aimed at different directions. Beamforming usually generates beams in multiple directions at the same time. Figure 2 shows the beam pattern or radiation pattern of a beamforming example. As shown in Figure 2, the beam having the strongest strength (i.e., the strongest beam) is called the Main Lobe (ML), others are called Side Lobes (SLs), and the strongest SL is called the Peak Side Lobe (PSL). It is desirable to aim the ML to the target direction in order to strengthen the ML, and weaken the SLs. This is because the ML delivers energy in the target direction, but SLs do not deliver energy in the target direction, which causes energy wastage.

This paper is to solve the antenna array beamforming optimization problem with the goal of minimizing the power ratio of PSL and ML aimed at a given target direction in order to increase energy efficiency. The optimization is applied to RF chargers for charging WRSN sensor nodes. It is assumed that an RF charger is equipped with a uniform circular array (UCA) antenna of the radius *λ* with 12 elements, where *λ* is the RF wavelength and elements are separated by a uniform distance of *λ*/$\sqrt{3}$ to prevent the mutual coupling effect [14]. One good property of the UCA is that it can produce nearly identical beam patterns for any target direction. Two evolutionary algorithms, namely the Evolutionary Beamforming Optimization (EBO) algorithm and the Evolutionary Beamforming Optimization Reseeding (EBO-R) algorithm, are proposed to solve the problem. The proposed algorithms are simulated for performance evaluation and are compared with a related algorithm, called Particle Swarm Optimization Gravitational Search Algorithm-Explore (PSOGSA-Explore) [15], to show their superiority. Note that the PSOGSA-Explore algorithm is for the collaborative beamforming optimization of antennas in random positions. It can be applied to UCA beamforming optimization when collaborative antennas are arranged in the form of UCA. Also note that the existing schemes proposed in [5,6,7,8,9,10] that use RF chargers are not compared with the proposed algorithms, as the algorithms can be applied to the schemes to improve their performance.

The rest of this paper is organized as follows. Section 2 describes some preliminary knowledge about antenna array beamforming and evolutionary algorithms. The PSOGSA-Explore algorithm [15] and existing related work [5,6,7,8,9,10] using RF chargers is also introduced in this section. Section 3 discusses assumptions and defines the problem to be solved. The details of the proposed algorithms are given in Section 4. Section 5 illustrates simulation and comparison results. Finally, Section 6 concludes this paper.

## 2. Background and Related Work

### 2.1. Antenna Array Beamforming

An antenna is an electrical device used to transmit or receive electromagnetic waves [13]. Specifically, an isotropic antenna is a theoretical antenna that radiates the same intensity of radiation in all directions. However, it does not exist in practice; it is only for the purpose of theoretical studies. In terms of the practical directional property, antennas can be divided into two main types: omnidirectional and directional. Omnidirectional antennas radiate energy almost equally in all directions. On the contrary, directional antennas have the directivity property whereby they radiate more energy in some preferred directions and radiate less in other directions.

An antenna array of multiple elements [13] can be applied to achieve higher directivity than that of a directional antenna. Every element’s activation magnitude and phase can be adjusted to form energy beams so that the array has good directivity. This is called antenna array beamforming. Antenna array beamforming can swiftly radiate most energy toward a target direction.

**Figure 3:**
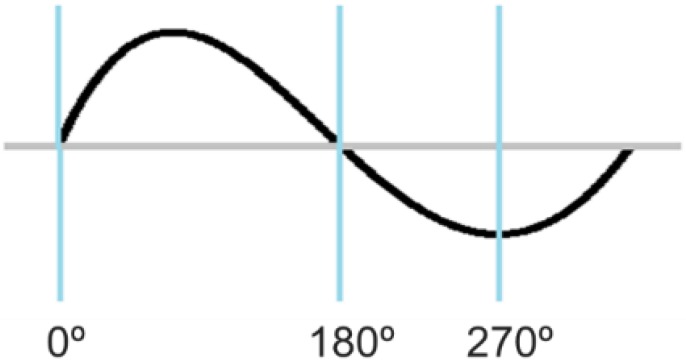
The phase of a waveform measured in degrees between 0° and 360°.

**Figure 4:**
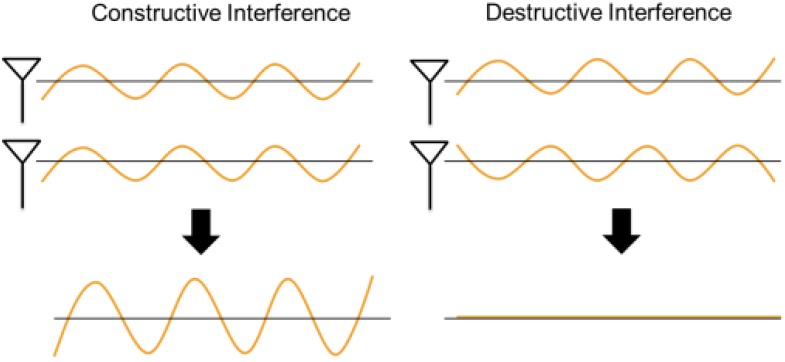
Constructive and destructive interference of waves.

**Figure 5:**
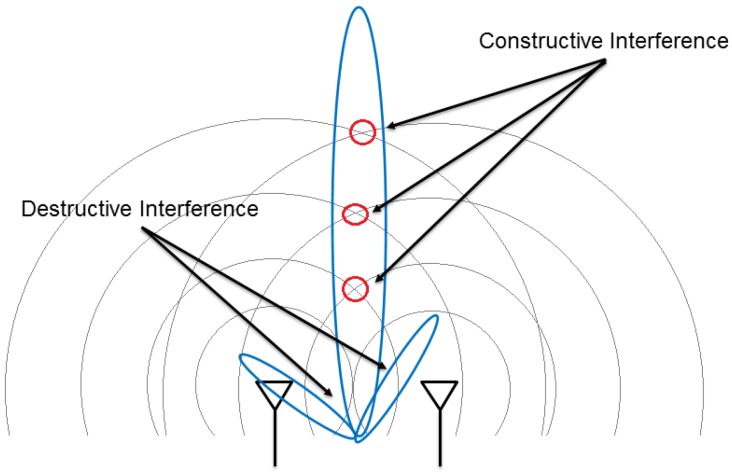
The beams caused by interference.

The phase of a radio wave, measured in degrees between 0° and 360°, is useful to describe a point in time on a waveform cycle, as shown in Figure 3. Two radio waves interfere with each other constructively or destructively to result in a new wave according to their phases. Figure 4 shows extreme examples of interference of two waves. On the one hand, when the peaks of the two waves coincide (i.e., their phases are aligned), the most constructive interference occurs. On the other hand, when the peak of one wave coincides with the trough of the other wave (i.e., their phases have a difference of 180°), the most destructive interference occurs. The phase of the signal fed to each antenna array element can be controlled for obtaining constructive interference in the target direction and destructive interference in other directions, as shown in Figure 5.

**Figure 6:**
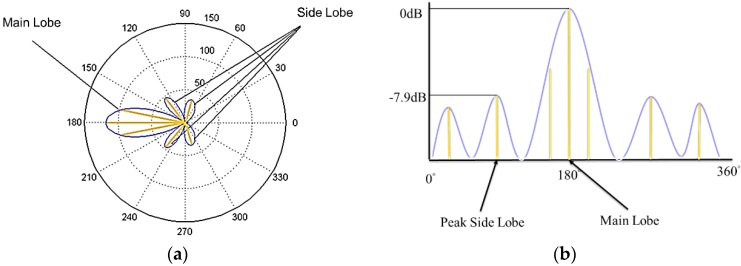
(**a**) The beam pattern of an antenna array beamforming, and (**b**) its associated Cartesian coordinate representation.

The circular distribution of signal strength associated with an antenna is called its radiation pattern or beam pattern, as shown in Figure 6a. The beam pattern can also be represented in the Cartesian coordinate form, as shown in Figure 6b, in which the strength of the main lobe is assumed to be 0 dB and side lobes are measured in the unit of dB relative to the main lobe. The antenna radiates most energy within the main lobe (ML), which contains the strongest signal, and radiates less energy within the side lobes (SLs). The SL that has the strongest signal is called the peak side lobe (PSL). By the definition, we can easily see the ML, SLs, and the PSL shown in Figure 6.

**Figure 7:**
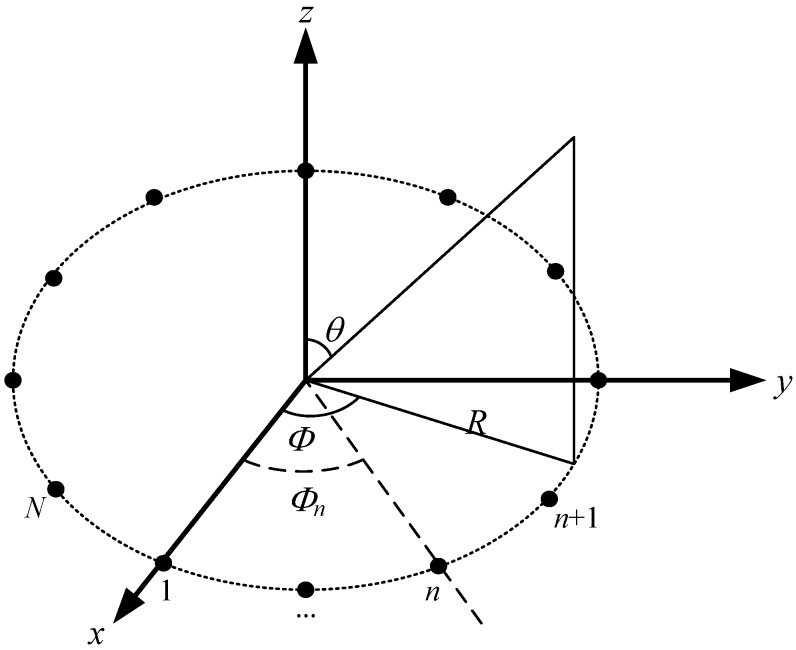
The geometric illustration of an *N*-element uniform circular array (UCA).

An array factor of an antenna array is a function that describes the beam pattern of the array [16]. Consider a UCA with *N* isotropic antenna elements that are equally spaced on the *x*-*y* plane on a circle of radius *R* (referring to Figure 7). Assume the weight (i.e., the excitation coefficient of the amplitude and the phase) and the azimuth of the *n*th element are *w_n_* and $\phi_{n}$, 1 ≤ *n* ≤ *N*, respectively. Let $w=\left(w_{1},\ldots ,\;w_{N}\right)$ be the vector of elements’ weights. The array factor of the UCA in the direction specified by the elevation θ and the azimuth ϕ is given by Equations (1) and (2) [15,16].

(1) $$\mathrm{AF}\left(\theta ,\;\phi ,\;w\right)=\overset{N}{\underset{n=1}{\sum }}w_{n}e^{j\left(\frac{2\pi }{\lambda }\right)R\sin\theta \;\cos\left(\phi -\phi_{n}\right)}$$

(2) $$w_{n}=\xi_{n}e^{j\rho_{n}}$$

Note that $w_{n}$ in Equation (2) is the weight (excitation coefficient) of the amplitude and the phase of the *n*th element, $\xi_{n}$ is the amplitude excitation of the *n*th element, and $\rho_{n}$ is the phase excitation of the *n*th element relative to the array center. This paper only considers the direction on the *x*-*y* plane, so we have θ = 90°. We can also omit the parameter θ and the term sin θ in Equation (1), as sin 90° = 1. We thus have

(3) $$\mathrm{AF}\left(\phi ,\;w\right)=\overset{N}{\underset{n=1}{\sum }}w_{n}e^{j\left(\frac{2\pi }{\lambda }\right)R\;\cos\left(\phi -\phi_{n}\right)}$$

By the array factor, the phase of the *n*th element can be calculated by Equation (4) to steer most of the energy in the direction specified by ϕ and θ=90°. Below, this is called phase calculation.

(4) $$\rho_{n}=-\frac{2\pi }{\lambda }\;R\;cos\left(\phi -\phi_{n}\right)$$

In Equation (4), $R\;\cos\left(\phi -\phi_{n}\right)$ is the projection of the distance between the *n*th element and the array center along the target direction. The projection is divided by the wavelength *λ* to derive how many times the projection is as large as *λ*. The divided projection is then multiplied by 2*π* to put the number of times in the unit of radians to represent the desired phase. Furthermore, a negative sign is added to the whole term to indicate that the element’s phase should shift earlier by the desired phase to align all element waves.

After deriving the phases of all UCA elements, the weight of elements depends solely on the elements’ amplitudes. Let ξ = ($\xi_{1},\;\ldots ,\xi_{N}$) be the vector of the amplitudes of the *N* elements. It is desirable to set up all *N* elements’ amplitudes properly to optimize the beam pattern of the UCA. There are many objectives of the beam pattern optimization. This paper adopts the objective of minimizing the normalized PSL relative to the ML in decibels, which is defined in Equation (5) as a function *f*(·).

(5) $$f\left(\phi ,\;w\right)=20\;log_{10}\frac{AF\;\left(\phi_{PSL},\;w\right)}{AF\;\left(\phi_{ML},\;w\right)}$$

In Equation (5), ϕ is the target direction, $\phi_{ML}$ indicates the direction of the ML, which is the same as ϕ, and $\phi_{PSL}$ indicates the direction of the PSL.

### 2.2. Evolutionary Algorithms

Evolutionary algorithms (EAs) use techniques that mimic biological evolution principles (e.g., the “survival of the fittest” principle) for solving optimization problems [17]. They are generally population-based, fitness-oriented, and variation-driven. An EA keeps a group of solution candidates for solving a given optimization problem, where a solution candidate is called an individual and the group is called a population. Every individual is represented by a special data structure (e.g., a chromosome of genes), which is associated with a fitness value for evaluating how close the individual is to the optimization goal. According to fitness values, individuals go through some variation operations (e.g., selection, reproduction, crossover, and mutation) to produce new generation of individuals with even better fitness values to survive. An EA has a loop of iterations or generations, which terminates when specific conditions (e.g., the maximum number of generations is reached) are made. Typical EAs include the genetic algorithm (GA), the evolution strategy (ES), and the genetic programming (GP). As our proposed algorithms are an ES algorithm, only the ES is elaborated below in this paper.

The evolution strategy (ES) was first proposed in the 1960s and further developed by Rechenberg and Schwefel in the 1970s [18,19]. For optimizing a goal, an ES algorithm keeps a population of individuals that are usually represented as real number vectors. A certain number of individuals are generated as the initial population in the first generation or iteration of the loop. A fitness value is associated with every individual for evaluating how close it is to the goal. All individuals in every generation are ranked according to their fitness values, and only the top few individuals are selected to produce offsprings mainly by mutation. An ES algorithm terminates only when certain conditions are met.

### 2.3. Particle Swarm Optimization for Beamforming

The particle swarm optimization (PSO) strategy [20], inspired by swarm intelligence, has basic concepts similar to those of evolutionary algorithms. It is also population-based, fitness-oriented, and variation-driven. Kennedy and Eberhart first introduced PSO [21] based on the foraging behavior of birds, as described below. Birds of a swarm seek food in a specific region. A bird can seek food on the basis of its own experience, as it keeps the local best known position of finding food. Birds are assumed to notify one another of food positions when they find food. Therefore, a bird can also seek food on the basis of the swarm experience, as it keeps the global best known position of finding food. In summary, a bird’s movement for seeking food is influenced by both its local best known food position and the global best known food position.

A PSO algorithm keeps a population of individuals. Each individual is called a particle, which represents a candidate solution, and is characterized by a position and a velocity in a *k*-dimension solution space. The goodness of a particle is evaluated on the basis of the fitness value of its position. A position has better fitness value if it is closer to the optimization goal. Initially, each particle has an arbitrary position and an arbitrary velocity. Iteration by iteration, a particle adjusts its velocity according to its current position, the local best position, and the global best position. It then updates its position by adding the velocity to the positon. Eventually, a near-optimal solution to the problem can be found. PSO algorithms are shown to be very useful for solving many optimization problems [22].

### 2.4. Related Work

In this subsection, related research results are described. They include a PSOGSA-E algorithm [15] for beamforming optimization, and the schemes [5,6,7,8,9,10] using RF chargers to charge WRSN sensor nodes, which can benefit from using antenna array beamforming due to the directivity property of beamforming. Below, we first describe the PSOGSA-E algorithm [15].

Jayaprakasam et al. proposed an algorithm, called Particle Swarm Optimization Gravitational Search Algorithm-Explore (PSOGSA-E) [15], for optimizing the beamforming beam pattern. To be specific, the optimization goal is to minimize the normalized PSL in decibels, as defined in Equation (5). The PSOGSA-E method is the combination of the PSO, the gravitational search algorithm (GSA), and the exploring concept to optimize the beamforming beam pattern. The PSOGSA-E method keeps a population of particles, and each particle moves to search for the optimal solution and influences each other in the solution space. The particles that have higher fitness values are assigned more mass in each iteration. Particles follow Newton’s law of universal gravitation to generate gravitation, further generate acceleration and velocity, and continuously update the optimal solution when exploring in the search space simultaneously. Jayaprakasam et al. confirmed through simulations that PSOGSA-E has better performance when compared with related algorithms, such as PSO, GSA, and PSOGSA.

Liao et al. [5] proposed two greedy algorithms, called the Node Based Greedy Cone Selection (NB-GCS) and the Pair Based Greedy Cone Selection (PB-GCS), to solve the wireless charger deployment optimization (WCDO) problem to deploy the minimum number of wireless RF chargers in a room needed to charge (cover) WRSN sensor nodes for fulfilling their energy requirements. The chargers are assumed to be deployed at grid points on a plan of fixed height, and they are assumed to be equipped with directional antenna having an effective charging region of a cone shape. The algorithms initially unmark all sensor nodes and then go through two stages. In the first stage, the NB-GCS algorithm generates cones (i.e., a charging region) on a “node by node” basis, while the PB-GCS algorithm generates cones on a “node-pair by node-pair” basis. In the second stage, both algorithms run iteration by iteration to greedily select the cone covering the most unmark sensor nodes. They mark every sensor node whose energy requirement is satisfied by newly selected cone, which corresponds to a charger with a specific direction antenna orientation. The two algorithms run until all of the sensor nodes are marked, which means their energy requirements are all satisfied. 

Chen et al. [6] proposed the PSCD (Particle Swarm Charger Deployment) algorithm using the PSO concept to solve the WCDO problem. Unlike previous solutions to the WCDO problem, the PSCD algorithm does not have the restriction that chargers must be deployed at grid point. Instead, they are assumed to be deployed at any point on the plan of a fixed height, which is a more practical assumption. The PSCD algorithm estimates the absorbed energy according to the distance and the angle between chargers and sensor nodes (i.e., harvesters). Based on the absorbed energy estimation, the PSCD algorithm relies on the PSO concept that uses the local optimum and global optimum to adjust locations and antenna directions of chargers to produce good solutions to the WCDO problem. The PSCD algorithm significantly outperforms the NB-GCS algorithm and the PB-GCS algorithm.

Jiang et al. [7] proposed the greedy cone covering (GCC) algorithm and the adaptive cone covering (ACC) algorithm to improve the NB-GCS algorithm and the PB-GCS algorithm by greedily and adaptively generating cones to cover as many as possible sensor nodes. The authors performed extensive experiments for evaluating the energy absorbed by the harvester for any given distance of horizontal angles (azimuths) or vertical angles (elevations) between the charger and the harvester (sensor). More energy consumption considerations and grid point separation analysis are included in Jiang et al.’s research.

Moraes et al. [8,9] proposed two hybrid-charging schemes that combine charging by a mobile charger and energy trading (or sharing) between neighboring sensor nodes. One scheme uses the mobile charger equipped with a directional antenna; the other scheme, an omnidirectional antenna. The scheme’s first partition sensor divides nodes into clusters, each of which has a cluster head and several cluster members. The schemes then go through two stages. In the first stage, each cluster is overcharged by the mobile charger, while in the second stage, the cluster head and other overcharged sensor nodes help charge undercharged sensor nodes in order for them to reach a target energy level. The proposed schemes have a shorter charging time than the scheme proposed by Fu et al., which enables all of the sensor nodes to reach the target energy level [10]. This is because Fu et al.’s scheme uses a mobile charger equipped with an omnidirectional antenna and plans optimal charger stops for the mobile charger to directly charge all sensor nodes, rather than only cluster heads, to reach the goal of minimizing the charging time.

## 3. Problem Definition

Consider a WRSN containing sensor nodes that are uniformly distributed over a specific field, and each of which is equipped with an RF harvester of an omnidirectional antenna. Every sensor node knows it position, and will send out its charging demand with its position when out of energy. RF chargers with known positions are deployed over the WRSN field; every RF charger is equipped with a UCA to form RF beams to charge sensor nodes. 

**Figure 8:**
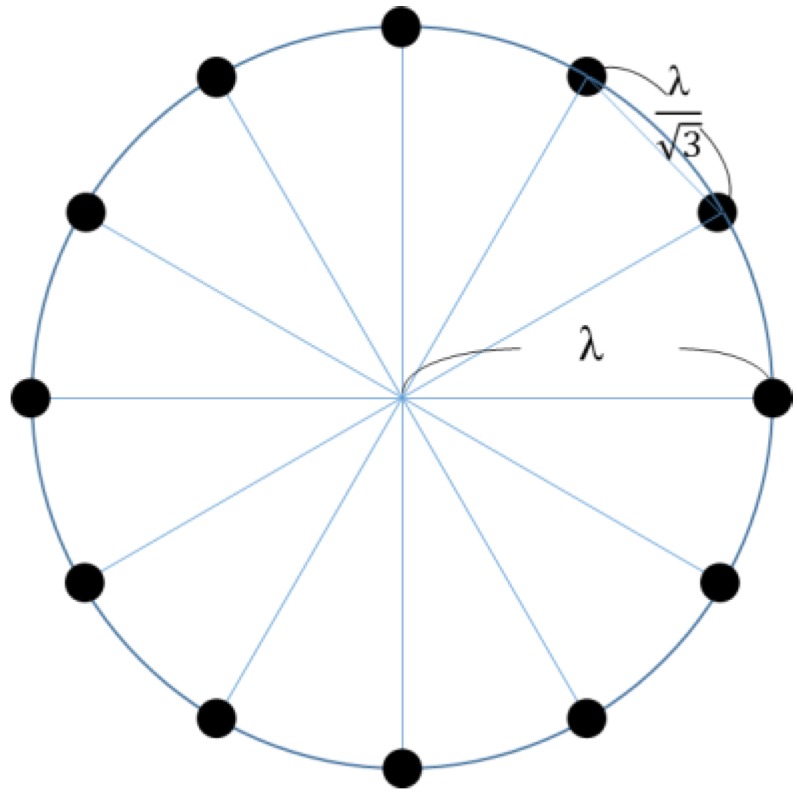
A UCA with the radius *λ* consisting of 12 elements separated by *λ*/$\sqrt{3}$.

A UCA consists of 12 isotropic elements and has the radius *λ*, which is the RF wavelength (referring to Figure 8). The elements are separated by a uniform distance of *λ*/$\sqrt{3}$. The separation setting is because the mutual coupling effect can be mitigated significantly for an element separation larger than *λ*/2. We can increase the circle radius to arrange more elements to have better beam patterns, as more elements usually leads to better beam patterns. However, the ML beam width increases with the circle radius. When the circle radius is *λ*, the ML beam width is 30°, which is satisfied. This is why we set the UCA radius to be *λ*.

.

It is assumed that a sensor node is within the effective charging area of at least one charger, and one of the chargers is elected somehow to charge the sensor node. With the positions of the sensor node and the charger, the UCA target direction can be derived. It is specified by the azimuth (angle) between the *x*-axis and the line going from the charger to the sensor node.

The problem to be solved in this paper is defined as follows. For a given target direction, it is the goal to steer the UCA for radiating the most energy in the target direction and much less energy in other directions. The goal is achieved by properly setting the elements’ excitation amplitudes, denoted by $\left(\xi_{1},\;\xi_{2},\;\xi_{3},\;\ldots ,\;\xi_{12}\right)$, with UCA elements’ excitation phases being set according to the phase calculation, as defined in Equation (4). Formally, the goal is formulated as minimizing the normalized PSL relative to the ML in decibels, as defined in Equation (5).

## 4. Proposed Algorithms for Beamforming Optimization

This section describe the two proposed EAs using ES, namely the Evolutionary Beamforming Optimization (EBO) algorithm and the Evolutionary Beamforming Optimization Reseeding (EBO-R) algorithm, to solve the problem defined in Section 3. The two algorithms are elaborated in the following subsections.

### 4.1. EBO Algorithm

The EBO algorithm keeps a population of *m* individuals, each of which is represented by an activation amplitude vector ξ = $\left(\xi_{1},\ldots ,\;\xi_{N}\right)$, where $0\leq \xi_{n}\leq$ 1 for 1≤n≤ *N* = 12. To be more precise, each individual is a vector of 12 real numbers between 0 and 1 to represent the activation amplitudes of 12 UCA elements. Given a target direction, the activation amplitude vector is taken as an argument to calculate the fitness value by Equations (1)–(5). Note that the fitness value is actual the normalized PSL in decibels relative to the ML. Therefore, the lower the fitness value, the better is the individual. The EBO algorithm takes the best *k* (say *k* = 3) individuals to perform the mutation operation for producing offspring of the new generation to replace the worst *k* individuals. The mutation operation for an individual is to substitute an arbitrary real number in the amplitude vector with a random real number between 0 and 1. The EBO algorithm continues generation by generation until the maximum generation is reached. 

Below is the pseudo code of the EBO algorithm (Algorithm 1).

**Algorithm 1:** Evolutionary Beamforming Optimization (EBO)**Input:** A UCA of *N* elements, a target direction ϕ in the *x*-*y* plane, the number *m* of individuals, the number *k* of individuals to be replaced, and the maximum generation *G_max_*
**Output:** Best amplitude vector ξ = $\left(\xi_{1},\ldots ,\;\xi_{N}\right)$, where $0\leq \xi_{n}\leq$ 1, and its normalized PSL η in decibels **Step 1:** Randomly generate *m* individuals $\left(\xi_{1}^{1},\ldots ,\;\xi_{N}^{1}\right)$,…,$\left(\xi_{1}^{m},\ldots ,\;\xi_{N}^{m}\right)$ as the initial population.**Step 2:** Calculate the fitness values for all individuals; sort them according to ascending fitness values.**Step 3:** Select the best *k* individuals to generate *k* mutant offsprings for replacing the worst *k* individuals to form the population of the next generation. For an individual *i* = $\left(\xi_{1}^{i},\ldots ,\;\xi_{N}^{i}\right)$, its mutant offspring is produced by performing $\xi_{j}^{i}$ = *rand*(0,1), where *j* is an arbitrary integer, 1≤j≤
*N*, and *rand*(0,1) is an arbitrary real number, 0 < *Rand*(0,1) ≤ 1. **Step 4:** Calculate the fitness values for the *k* offsprings, and re-sort all individuals.**Step 5:** If the max generation *G_max_* is not reached, then go to **Step 3.****Step 6:** Output the amplitude vector ξ  = $\left(\xi_{1},\ldots ,\;\xi_{N}\right)$ of the best individual and its normalized PSL η.

### 4.2. EBO-R Algorithm

By observing several simulation experiments of the EBO algorithm, we found that most of the best individuals in the current generation are mutated from the best individuals in the previous generation. Moreover, the best individual at the end of the experiment usually has a high probability to be the offspring of one of the best *k* individuals in the initial population. That is because the EBO algorithm exploits good results associated with the individuals in the previous generation for the purpose of improving the results of the current generation. Therefore, it is likely that the final result returned by the EBO algorithm is not optimal or even not good enough if the initial individuals are not good enough. In summary, the EBO algorithm should be improved by adding more exploration capability to search for better solutions.

The EBO-R algorithm involves a reseeding step to improve the EBO algorithm. In each generation, *h* random individuals are generated to replace the worst *h* individuals in the current generation. This is similar to reseeding a field with a number of new species of plants (i.e., random individuals) for the hope that some of the new species of plants are better than all current plants. This is why the improved algorithm is called the evolutionary beamforming optimization reseeding (EBO-R) algorithm. Although we can increase the initial population size to increase the EBO algorithm’s exploration capability, computation increases significantly with the population size. On the contrary, the EBO-R algorithm only generates *h* individuals and remove *h* individuals in every generation. In such a manner, the exploration capability is increased significantly without causing too much computation, because the population size is not increased at all.

Below is the pseudo code of the EBO-R algorithm (Algorithm 2).

**Algorithm 2:** Evolutionary Beamforming Optimization Reseeding (EBO-R)**Input:** A UCA of *N* elements, a target direction ϕ in the *x-y* plane, the number *m* of individuals, the number *k* of individuals to be replaced, the number *h* of reseeded individuals, and the maximum generation *G_max_***Output:** Best amplitude vector ξ  = $\left(\xi_{1},\ldots ,\;\xi_{N}\right)$, where $0\leq \xi_{n}\leq$1, and its normalized PSL η in decibels **Step 1:** Randomly generate *m* individuals $\left(\xi_{1}^{1},\ldots ,\;\xi_{N}^{1}\right)$,…,$\left(\xi_{1}^{m},\ldots ,\;\xi_{N}^{m}\right)$ as the initial population.**Step 2:** Calculate fitness values for all individuals; sort them according to ascending fitness values.**Step 3:** Select the best *k* individuals to generate *k* mutant offsprings for replacing the worst *k* individuals; resort all individuals. For an individual *i* = $\left(\xi_{1}^{i},\ldots ,\;\xi_{N}^{i}\right)$, its mutant offspring is produced by performing $\xi_{j}^{i}$ = *rand*(0,1), where *j* is an arbitrary integer, 1≤j≤
*N*, and *rand*(0,1) is an arbitrary real number, 0 < *Rand*(0,1) ≤ 1.**Step 4:** Randomly generate *h* individuals for replacing the worst *h* individuals to form the population of the next generation.**Step 5:** Calculate fitness values for the (*k + h*) new offsprings and re-sort all individuals.**Step 6:** If the max generation *G_max_* is not reached, then go to **Step 3.****Step 7:** Output the amplitude vector ξ = $\left(\xi_{1},\ldots ,\;\xi_{N}\right)$ of the best individual and its normalized PSL η.

## 5. Simulation and Analysis

In this section, the simulation results of the proposed EBO and EBO-R algorithms are compared with those of the related PSOGSA-E algorithm in terms of (1) the beamforming performance for a UCA to be aimed at a given direction, (2) the convergence speed, (3) the stability, and (4) the run time. The UCA has the radius *λ* which consists of 12 isotropic antennas separated by a uniform distance of *λ*/$\sqrt{3}$, where *λ* is the RF wavelength. The performance is measured by the fitness value of the normalized PSL in decibels, as defined in Equation (5). The basic UCA case in which all antennas have the maximum activation amplitude (normalized as 1) is also compared. 

The parameter settings of the proposed EBO and EBO-R algorithms are as follows. The population size *m* is set as 100. The number *k* of individuals to be replaced is set as 3, the number *h* of reseeded individuals is set as 10. The maximum generation *G_max_* is set as 20, 50 or 100. Simulation experiments are executed 100 times for each case, and the best experiment result is taken as the representative result of the case.

**Figure 9:**
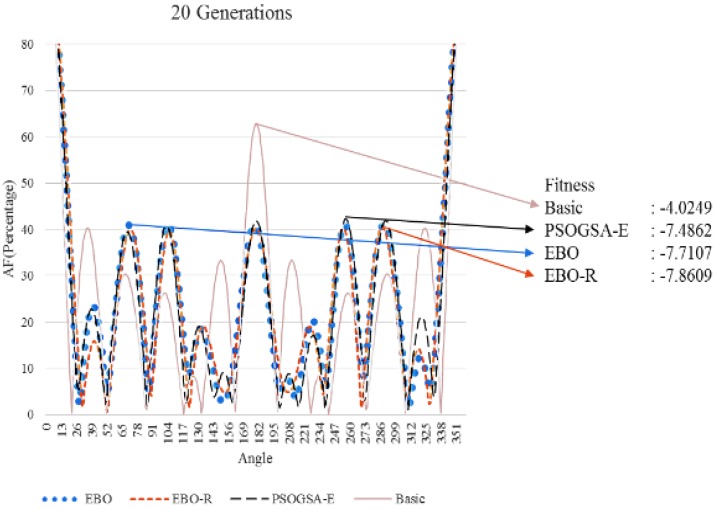
Beam patterns of different algorithms (20 generations).

**Figure 10:**
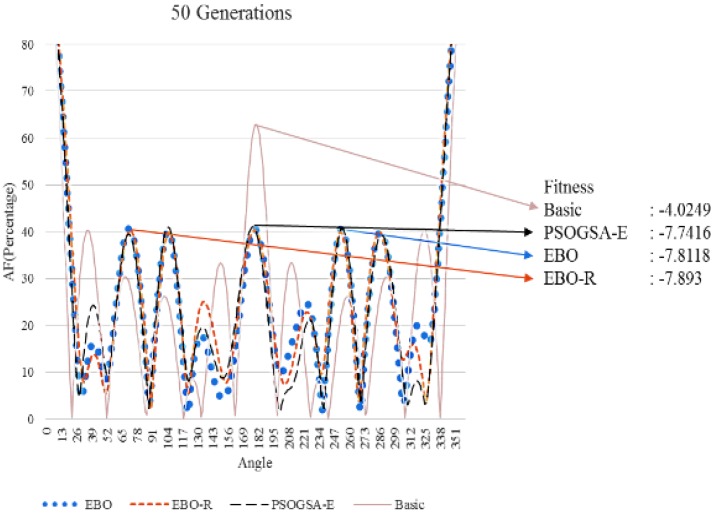
Beam patterns of different algorithms (50 generations).

**Figure 11:**
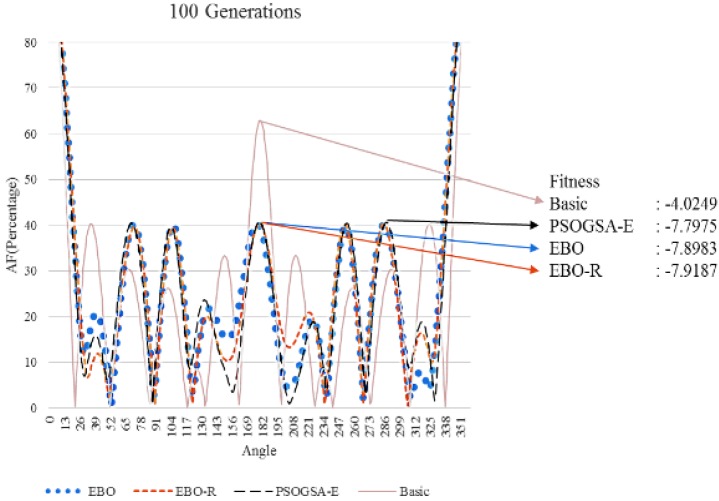
Beam patterns of different algorithms (100 generations).

The beam patterns of the simulation results are shown in Figure 9, Figure 10 and Figure 11. Note that the vertical axis in the figures represents the percentage (ratio) of the wave strength over the strength of the ML. By the figures, we can observe that the three algorithms have much better fitness (i.e., normalized PSL in decibels) than the basic case. We can also observe that the superiority of the algorithms over the basic case increases with the maximum generation. Among the three algorithms, the EBO-R algorithm is the best, the EBO algorithm is the mediate, and the PSOGSA-E is the worst in terms of the normalized PSL in decibels. Note that size lobe may appear in different angles (directions) for experiments running for different generations. Also note that although the fitness results are similar for the cases of 20, 50, and 100 generations; we still demonstrate all figures of the three cases to show that proposed algorithm converges fast, i.e., all cases converge to good enough fitness even for small number of generations.

**Figure 12:**
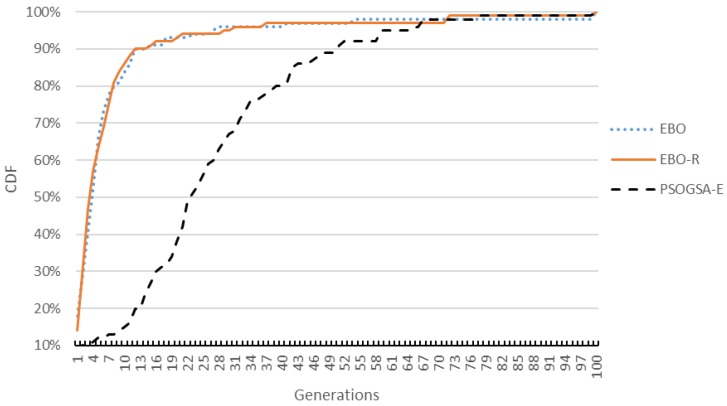
Cumulative distribution function (CDF) comparisons of different algorithms.

Figure 12 further shows convergence speed comparisons for the EBO, the EBO-R, and the PSOGSA-E algorithms. We run the three algorithms for 100 generations and stop them at a generation when the fitness value (i.e., normalized PSL in decibels) reaches −6.7, which is the average fitness of 100 experiments of running the PSOGSA-E algorithm for 20 generations. Every algorithm is run 100 times to generate the cumulative distribution function (CDF). By Figure 12, we can observe that the EBO and the EBO-R algorithms converge fast, they have very similar CDF to reach the fitness of −6.7, and they both significantly outperform the PSOGSA algorithm.

As stated earlier, simulation experiments are executed 100 times for a case, and the best result is taken as the representative result of the case. Table 1, Table 2 and Table 3 show the best, the average, the worst, and the standard deviation of the experiment results for the EBO, the EBO-R, and the PSOGSA-E algorithms. The standard deviation can be used to measure the stability of the algorithms. As shown in the tables, the EBO-R algorithm is the best, the EBO algorithm is the mediate, and the PSOGSA-E algorithm is the worst in terms of stability. That is to say, the EBO-R, the EBO and the PSOGSA-E algorithms have, respectively, the least, mediate, and the largest amount of variation of output results. Therefore, the EBO-R algorithm is the most stable, and we might run fewer experiments for it to find good results, as its experiment’s output results are close to the average value.

**Figure 13:**
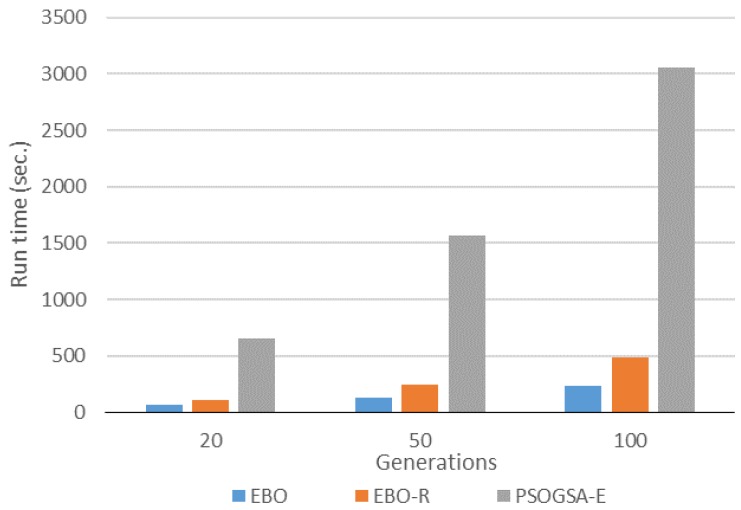
Run time comparisons of different algorithms.

Figure 13 shows the run time for the EBO, the EBO-R, and the PSOGSA-E algorithms. By Figure 13, we can observe that the run time increases with the number of generations. The EBO algorithm has the shortest run time, the EBO-R algorithm has the mediate run time, and the PSOGSA-E algorithm has the longest run time.

In summary, the EBO-R has the best performance in the aspects of the normalized PSL in regards to decibels and stability, while the EBO algorithm has the best performance in regards to run time. The EBO algorithm and the EBO-R algorithm have similar performance in regards to the convergence speed. They both are much better than the PSOGSA-E algorithm in all aspects.

## 6. Conclusions

This paper proposes two EAs to solve the beamforming optimization problem with the goal to minimize the normalized PSL in decibels for a UCA. The UCA is attached to an RF charger to charge a WRSN sensor node situated in a given target direction relative to the charger. The UCA has a radius *λ* consisting of 12 isotropic antennas, where *λ* is the RF wavelength.

The proposed algorithms, called the Evolutionary Beamforming Optimization (EBO) algorithm and the Evolutionary Beamforming Optimization Reseeding (EBO-R) algorithm, use the ES to solve the problem. According to the simulation results, the two algorithms are better than a related PSO-based algorithm, called PSOGSA-E [15], in the aspects of the normalized PSL, stability, convergence speed, and run time. The EBO-R algorithm is better than the EBO algorithm in the first two aspects, and the two algorithms have similar convergence speed. However, the EBO has a shorter run time. Therefore, both algorithms have their own merits to be used in different scenarios.

In the future, we plan to apply more mechanisms to solve the beamforming optimization problem. For example, we will try to combine the concepts of the ES and PSO to design algorithms for solving the problem. Moreover, we also plan to include more objectives in the problem. For example, besides minimizing the normalized PSL, we will try to add maximizing the charging distance and maximizing the directivity as other objectives. The problem thus becomes a multi-objective optimization problem, which needs more complicated algorithms to solve. We also plan to investigate the harvesting efficiency of multiple rechargeable sensor nodes with respect to one or multiple UCA-based chargers by using the proposed algorithms and the harvesting model proposed in [23,24]. For future work, we also intend to apply the proposed algorithms to charger deployment studied in [5,6,7] and mobile chargers used in [8,9,10] for achieving better performance.

## Figures and Tables

**Table 1:** Statistics for different algorithm (20 generations).

20 Generations			
	EBO	EBO-R	PSOGSA-E
Best	−7.71072	−7.860932947	−7.486170973
Average	−7.170361633	−7.272883095	−6.677645065
Worst	−6.399863587	−6.143560656	−5.708761616
Std dev.	0.319849064	0.314264741	0.349424293

**Table 2:** Statistics for different algorithm (50 generations).

50 Generations			
	EBO	EBO-R	PSOGSA-E
Best	−7.811796032	−7.892972282	−7.741563549
Average	−7.285225389	−7.381858066	−7.073319002
Worst	−6.19788195	−6.726870427	−6.321852079
Std dev.	0.317761699	0.27960251	0.331257561

**Table 3:** Statistics for different algorithms (100 generations).

100 Generations			
	EBO	EBO-R	PSOGSA-E
Best	−7.898287758	−7.918690025	−7.797466574
Average	−7.348459928	−7.45632991	−7.276442259
Worst	−6.504064398	−6.822299679	−6.574461271
Std dev.	0.33953628	0.261665257	0.267682804
